# Rescue Strategy in Cardiogenic Shock: Emergency Transcatheter Aortic Valve Implantation for Failed Bioprosthetic Valve – Case Report

**DOI:** 10.15388/Amed.2025.32.1.1

**Published:** 2025-02-18

**Authors:** Ricardas Kundelis, Vilhelmas Bajoras, Sigitas Čėsna, Giedrius Davidavičius

**Affiliations:** 1 Clinic of Cardiac and Vascular Diseases, Institute of Clinical Medicine, Faculty of Medicine, Vilnius University, Vilnius, Lithuania; 2 Vilnius University Hospital Santaros Klinikos, Vilnius, Lithuania

**Keywords:** TAVI, transcatheter aortic valve implantation, bioprosthetic valve failure, cardiogenic shock, TAVI, perkateterinis aortos vožtuvo implantavimas, biologinio vožtuvo nepakankamumas, kardiogeninis šokas

## Abstract

**Background:**

*Transcatheter aortic valve implantation* (TAVI) has emerged as an essential therapeutic intervention for patients with severe aortic valve disease, providing a less invasive alternative to traditional surgery, particularly in high-risk individuals. TAVI is also increasingly utilized as a valve-in-valve strategy in cases of a bioprosthetic valve failure. However, data on the efficacy of TAVI in the context of a hemodynamic collapse remain limited.

**Methods:**

This report represents a young, high-risk patient with a failed bioprosthetic valve and cardiogenic shock treated successfully with TAVI.

**Results:**

Emergency TAVI using a *Medtronic Evolut Pro+* device achieved rapid hemodynamic stabilization and favorable post-procedural clinical and functional outcomes. Intermittent complete atrioventricular block necessitated the implantation of a permanent pacemaker.

**Conclusions:**

This case highlights the potential of TAVI as a safe and effective intervention in critical clinical scenarios. Emergency TAVI is a viable therapeutic intervention for patients with failed surgical bioprostheses presenting with cardiogenic shock.

## Abstract (it)

**Įvadas:**

Perkateterinis aortos vožtuvo implantavimas (TAVI) tapo neatsiejama pacientų, kuriems nustatyta didelio laipsnio aortos vožtuvo patologija ir didelė operacinio gydymo rizika, minimaliai invazyvaus gydymo alternatyva. TAVI vis dažniau atliekamas esant implantuotų biologinių vožtuvų nepakankamumui. Vis dėlto duomenų apie TAVI efektyvumą esant nestabiliai hemodinamikai trūksta.

**Metodai:**

Šiame straipsnyje pristatomas sėkmingas jauno didelės rizikos paciento, kuriam nustatytas biologinio vožtuvo nepakankamumas, dėl kurio išsivystė kardiogeninis šokas, gydymo TAVI atvejis.

**Rezultatai:**

Neatidėliotinas TAVI naudojant *Medtronic Evolut Pro+* vožtuvą užtikrina greitą hemodinamikos stabilizavimą ir geras poprocedūrines klinikines ir funkcines baigtis. Dėl intermituojančios III laipsnio atrioventrikulinės blokados pacientui buvo implantuotas širdies stimuliatorius.

**Išvados:**

Šis atvejis leidžia patvirtinti TAVI saugumą ir efektyvumą esant kritinei paciento būklei. Neatidėliotinas TAVI gali būti atliekamas pacientams, kuriems nustatytas biologinio vožtuvo nepakankamumas ir dėl jo išsivystęs kardiogeninis šokas.

## Background

Over the past two decades, *Transcatheter Aortic Valve Implantation* (TAVI) has evolved into a well-established therapeutic option for patients with severe *aortic valve stenosis* (AS) [1]. Additionally, TAVI is increasingly used as a valve-in-valve solution for patients with failing bioprosthetic aortic valves by providing a minimally invasive alternative to repeat open-heart surgery, particularly for high-risk or elderly individuals [2]. Emerging data from single-center and small cohort studies suggest that an urgent or emergent TAVI may also be feasible and effective approach for patients with severe AS complicated by cardiogenic shock or acute decompensated heart failure [3–5]. Herein, we present the case of a young, high-risk patient presenting with a failed bioprosthetic valve and cardiogenic shock.

## Case presentation

A 54-year-old male, six years post-Bentall procedure with a Sorin Mitroflow 25 mm (*Sorin Group*, Saluggia, Italy) prosthetic valve, was admitted for redo surgery due to severe aortic valve insufficiency. His medical history included hypertension, dyslipidemia, and erosive gastritis. On physical examination, his blood pressure was 102/70 mmHg, and his heart rate was 96 beats per minute; a new systolic-diastolic murmur was noted in the aortic area, accompanied by clinical signs of congestion. His ongoing medications included beta blockers, oral diuretics, and lipid-lowering agents.

**Fig. 1:**
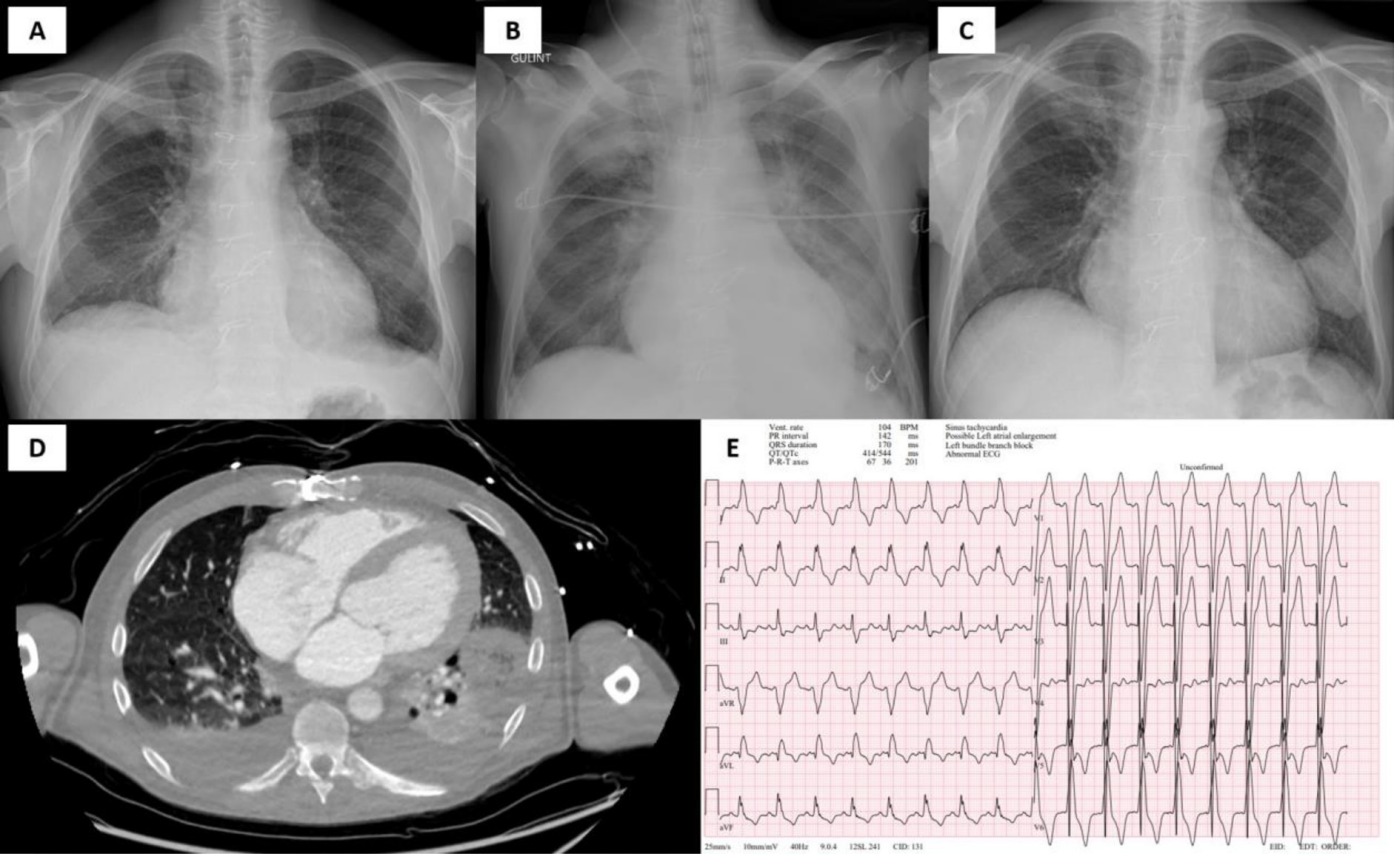
Patient initial diagnostic workup. **A, B** – chest radiograph demonstrating right upper lung infiltration and moderate pleural effusion on and during admission; **C** – significant improvement upon discharge; **D** – chest CT demonstrating the same right upper lung (S1–S2) infiltration and moderate pleural effusion; **E** – electrocardiogram demonstrating sinus tachycardia and left bundle branch block

The initial diagnostic workup revealed sinus tachycardia on ECG with a newly developed left bundle branch block [Fig. 1, E]. Laboratory findings showed moderate anemia, neutrophilic leucocytosis, and elevated C-reactive protein, suggesting an inflammatory or infectious etiology. Blood and urine cultures returned negative results. *B-type natriuretic peptide* (BNP) levels were significantly elevated at 4576 ng/L, consistent with severe heart failure, while D-dimer levels remained within the normal range. Chest imaging (X-ray and CT) showed infiltration in the right upper lung (S1–S2) and moderate pleural effusion [Fig. 1, A–B, D].

**Fig. 2:**
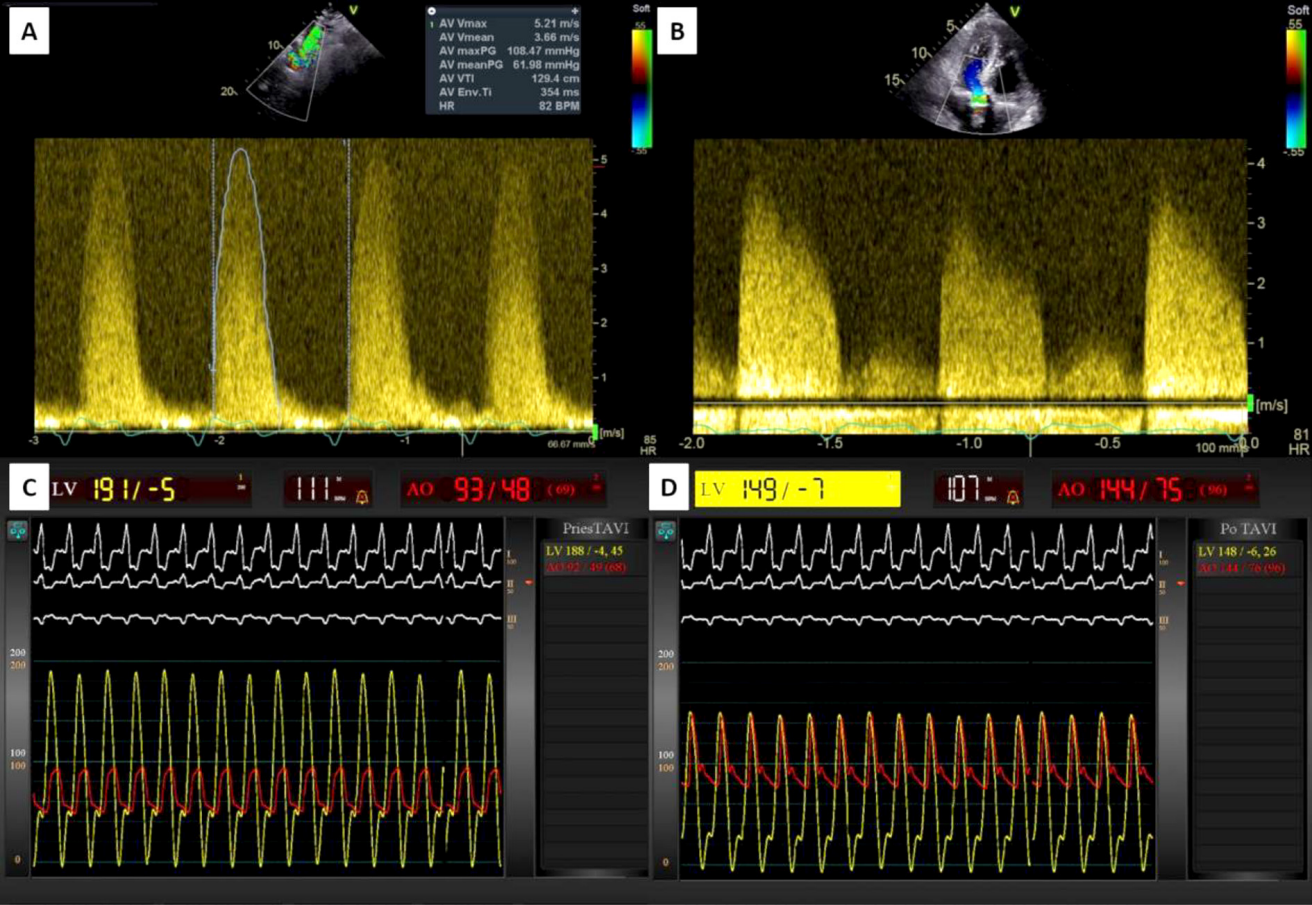
Patient hemodynamic characteristics pre- and post-TAVI. **A** – transoesophageal echocardiography demonstrating severe aortic valve stenosis on continuous-wave Doppler; **B** – transthoracic echocardiography demonstrating concomitant aortic regurgitation; **C** and **D** – aortic and ventricular pressure curves pre- and post-TAVI

*Transthoracic echocardiography* (TTE) demonstrated eccentric left ventricular hypertrophy with a reduced left ventricular ejection fraction (LVEF) of 33%. Severe aortic stenosis was observed, with a mean gradient of 62 mmHg and an aortic valve area of 0.72 cm^2^, accompanied by aortic regurgitation. Postcapillary pulmonary hypertension was present, with a systolic pulmonary artery pressure of 66 mmHg and pulmonary vascular resistance measured at 9.7 Wood units [Fig. 2, A and B; Supplementary Video 1]. *Transoesophageal echocardiography* (TOE) showed no valvular or periprosthetic *infective endocarditis* (IE).

Video 1

The differential diagnosis for the patient included IE, bioprosthetic valve thrombosis, or degeneration, complicated with worsening heart failure. At the time of admission, the patient did not meet the criteria for a definitive IE diagnosis, according to the *2023 European Society of Cardiology Guidelines*, as only two minor criteria were fulfilled: the presence of predisposing conditions and possible pulmonary infarcts [6]. TTE and TOE were inconclusive in differentiating between bioprosthetic thrombosis and degeneration. Hence, anticoagulation therapy was not initiated. The primary working diagnosis remained a failed bioprosthetic valve.

## Management and outcome

Despite optimal medical therapy, the patient’s condition rapidly deteriorated, leading to cardiac arrest. Following successful resuscitation, an emergency *transcatheter aortic valve implantation* (TAVI) was performed using a *Medtronic Evolut^TM^ PRO+* 26 mm valve (*Medtronic*, Minneapolis, MN, USA). Invasive hemodynamics assessment revealed a peak-to-peak systolic gradient of 96 mmHg across the aortic bioprosthesis. The procedure, supported by cerebral embolic protection, resulted in immediate hemodynamic improvement [Fig. 2, C–D; Supplementary Video 2].

Video 2

In the postoperative period, the patient developed a femoral artery pseudoaneurysm, which was managed conservatively. A Klebsiella pneumoniae infection was identified and treated with meropenem. The patient experienced a favorable recovery, becoming asymptomatic, and was discharged 20 days post-TAVI with a significant improvement in functional capacity. TTE (see Supplementary Video 3) demonstrated a normal prosthetic valve function at a three-month follow-up, an improved LVEF of over 55%, a slightly reduced global longitudinal strain (-14.8%), and no signs of pulmonary hypertension. However, Holter ECG monitoring revealed intermittent complete atrioventricular block, necessitating the implantation of a permanent pacemaker.

Video 3

## Discussion

This is the first case to illustrate the role of emergency valve-in-valve TAVI in managing high-risk patients with failed bioprosthetic valves with cardiogenic shock. Traditionally, the redo open-heart surgery is considered the gold standard for managing such cases. However, the surgical risk is significantly elevated in patients with compromised hemodynamic stability, as reflected in this patient’s *EuroSCORE II* of 19.92%.

As demonstrated in this case, the use of TAVI in patients presenting with cardiogenic shock underscores the procedure’s ability to achieve rapid hemodynamic stabilization. While previous reports have indicated the potential effectiveness of TAVI in critically ill patients, emergency TAVI remains a relatively rare and technically challenging intervention. In this case, the decision to perform TAVI was driven by the urgency of the clinical situation, as medical management alone was insufficient to stabilize the patient.

## Conclusion

In conclusion, emergency TAVI is an effective and safe therapeutic intervention for patients with failed surgical bioprostheses complicated by cardiogenic shock. It highlights the significant hemodynamic improvement and stabilization post-TAVI. This case exemplifies the lifesaving potential of emergency TAVI for critically ill or high-risk patients, offering a significant enhancement in prognosis and the quality of life, positioning TAVI as a favorable and efficient treatment strategy for challenging cardiac cases.

## Data Availability

The authors confirm that the data supporting the findings of this study are available in the article.
